# Human Coxsackie- and adenovirus receptor is a putative target of neutrophil elastase-mediated shedding

**DOI:** 10.1007/s11033-022-07153-2

**Published:** 2022-02-05

**Authors:** Leonie Herrmann, Louise Schelletter, Raimund Hoffrogge, Karsten Niehaus, Volker Rudolph, Martin Farr

**Affiliations:** 1 Clinic for General and Interventional Cardiology/Angiology, Herz- und Diabeteszentrum NRW, Ruhr-Universität Bochum, Georgstr. 11, 32545 Bad Oeynhausen, Germany; 2 Proteome and Metabolome Research, Center for Biotechnology (CeBiTec), Faculty of Biology, Bielefeld University, Universitätsstr. 25, 33615 Bielefeld, Germany; 3 Cell Culture Technology, Technical Faculty, Bielefeld University, Universitätsstr. 25, 33615 Bielefeld, Germany

**Keywords:** Coxsackie- and adenovirus receptor, Shedding, Neutrophil elastase, Proteolysis

## Abstract

**Background:**

During viral-induced myocarditis, immune cells migrate towards the site of infection and secrete proteases, which in turn can act as sheddases by cleaving extracellular domains of transmembrane proteins. We were interested in the shedding of the Coxsackie- and adenovirus receptor (CAR) that acts as an entry receptor for both eponymous viruses, which cause myocarditis. CAR shedding by secreted immune proteases could result in a favourable outcome of myocarditis as CAR’s extracellular domain would be removed from the cardiomyocytes’ surface leading to decreased susceptibility to ongoing viral infections.

**Methods and results:**

In this work, matrix metalloproteinases and serine proteinases were screened for their proteolytic activity towards human CAR. Whereas matrix metalloproteinases, proteinase 3, and cathepsin G did not cleave human recombinant CAR or only within long incubation times, neutrophil elastase showed a distinct cleavage pattern of CAR’s extracellular domain that was time- and dose-dependent. Neutrophil elastase cleaves CAR at its membrane-proximal immunoglobulin domain as we determined by nanoLC-MS/MS. Furthermore, neutrophil elastase treatment of cells reduced CAR surface levels as seen by flow cytometry and immunofluorescence microscopy.

**Conclusions:**

With this study, we show that CAR might be a target for shedding by neutrophil elastase.

**Supplementary Information:**

The online version contains supplementary material available at 10.1007/s11033-022-07153-2.

## Introduction

The Coxsackie- and adenovirus receptor (CAR) is a transmembrane protein with two extracellular immunoglobulin domains. CAR plays an important role during embryogenesis as it is crucial for the development of the heart [1] and the lymphatic vasculature system [2]. It was first described as entry receptor for both human pathogen viruses [3, 4]. Viruses bind to CAR’s membrane-distal D1 immunoglobulin domain that also forms homodimers.

Coxsackieviruses and adenoviruses cause a variety of diseases by infecting many tissues and organs including myocardium, pericardium, brain, pancreas, and gastrointestinal and respiratory tracts [5, 6]. Tissue injury due to viral infections leads to an inflammatory response of the host’s immune system. Immune cells are recruited and secrete proteases in order to migrate to the site of infection [7, 8]. Matrix metalloproteinases (MMPs) as well as serine proteinases like cathepsin G (CG), proteinase 3 (PR3), and neutrophil elastase (NE) in neutrophil granules exhibit strong proteolytic activity when secreted [9]. Several transmembrane proteins located in cell–cell contacts are proteolytically processed by neutrophil proteases, e.g. E-cadherin, ICAM-1, PECAM-1, and NCAM [10–14].

Therefore, the question was whether CAR’s extracellular domain is a target of neutrophil proteases. Defined cleavage of the extracellular domain of a transmembrane protein that results in the release of a soluble fragment is called shedding [15]. CAR shedding would limit ongoing virus infections by removing virus receptors from the cell surface. CAR surface expression levels in heart and pancreas correlate with the tissues’ susceptibility to virus infections [16, 17]. Furthermore, soluble CAR extracellular domain would function as a virus trap as was shown for CAR recombinant proteins in vitro and in vivo [18–20].

Murine CAR is shed by membranous A disintegrin and metalloproteinase 10 and 17 (ADAM10 and ADAM17) [21, 22]. Here we investigated, if human CAR is proteolytically processed by soluble proteases that are secreted by immune cells during virus-induced inflammation.

## Materials and methods

### Material and cell lines

CAR recombinant human extracellular domain (rhECD, amino acids 20-237 with C-terminal 6xHis tag) was purchased from abcam (Cambridge, UK, Cat.-No. ab168893) and reconstituted in sterile deionised water. Human neutrophil elastase (NE) from blood cells was obtained from Merck (Darmstadt, Germany; Cat.-No. 324681) and resuspended in NE reconstitution buffer (50 mM sodium acetate, 200 mM NaCl, pH 5.5). Human cathepsin G (CG) from neutrophils was obtained from Enzo (Farmingdale, NY, USA; Cat.-No. BML-SE283) and resuspended in CG reconstitution buffer (50 mM sodium acetate, 150 nM NaCl, pH 5.5). Human proteinase 3 (PR3) from neutrophils (Enzo; Cat.-No. BML-SE498) was resuspended in deionised water. Catalytic domains of human matrix metalloproteinases (MMP-1, -2, -3, -7, -8, -9, -10, -11, -12, and -13) were purchased from Enzo. General activity and specifity of proteases were tested with standard artificial substrates and protease inhibitors (phenylmethylsulfonyl fluoride (PMSF) and ethylenediaminetetraacetic acid (EDTA)).

Compositions of assay buffers were: NE assay buffer (100 mM Tris–HCl, 500 mM NaCl, pH 7.5); CG assay buffer (160 mM Tris–HCl, 1.6 M NaCl, pH 7.4); PR3 assay buffer (100 mM MOPS, 500 mM NaCl, pH 7.5); MMP assay buffer (50 mM HEPES, 10 mM CaCl_2_, 0.05% Brij-35, pH 7.0). For MMP-3, pH was adjusted to 6.0.

Three different anti-CAR antibodies were used in this study: Cat.-No. ab189216 from abcam was used for Western blotting, Cat.-No. sc-56892 from Santa cruz was used in flow cytometry, and Cat.-No. 05644 from Merck was used for immunofluorescence staining.

The monoclonal Chinese hamster ovary cell line expressing human CAR (CHO-CAR [23]) was grown in Ham’s F12 with 200 µg/ml Zeocin™ (InvivoGen, Toulouse, France). Human cell lines HeLa (cervix carcinoma) and SW13 (adrenal gland carcinoma) were grown in DMEM. Both media were supplemented with penicillin (100 U/mL), streptomycin (100 µg/ml), amphotericin B (0.25 µg/ml), and 10% FBS.

### Digestion of rhECD with proteases and deglycosylation with PNGase F

CAR rhECD was mixed with proteases in assay buffers and incubated at 37 °C (concentrations and incubation times varied as indicated in the text). For undigested control samples, reconstitution buffer was added instead of protease. Samples were either directly used for PAGE analysis, or treated with PNGase F (NEB, Ipswich, MA, USA, Cat.-No. P0710S) according to the manufacturer’s protocol.

General activity of proteases was confirmed using chromogenic and fluorescent substrates according to the manufactures’ protocols. Signals were detected using a TECAN microplate reader (Männedorf, Switzerland). Substrate for NE and PR3 was from Merck (Cat.-No. 324696). CG substrate was purchased from Enzo (Cat.-No. BML-P141). MMP-3 and MMP-10 activity was tested with fluorescent substrate II from R&D systems (Minneapolis, MN, USA; Cat.-No. ES002). All other MMPs cleave fluorescent substrate IX from R&D Systems (Cat.-No. ES010).

### PAGE, colloidal blue staining, silver staining, and Western blot

PAGE and gel staining were performed with materials from Invitrogen, Thermo Fisher Scientific, Waltham, MA, USA. Digestion samples of CAR rhECD were mixed with lithium dodecyl sulfate (LDS) sample buffer (Cat.-No. NP0007) including 50 mM DTT and heated for 5 min at 70 °C. Samples were loaded onto a 4–12% Bis–Tris gel (Cat.-No. NP0323) and the gel run was performed for 30 min at 200 V. Running buffer was MES buffer (Cat.-No. NP0002) with antioxidant (Cat.-No. NP0005). The gel was either stained with the colloidal blue kit (Cat.-No. LC6025) or the silver stain kit (Cat.-No. 24612) according to the manufacturer’s protocols. Colloidal blue staining required 1 µg of rhECD per lane. For silver staining and Western blot, 100 ng were loaded per lane.

Estimation of molecular masses of cleavage products in a silver-stained gel was performed with LabImage software (Kapelan Bio-Imaging, Leipzig, Germany).

For Western blotting, proteins were transferred to a PVDF membrane (Cat.-No. LC2002) for 1 h at 160 mA. Membrane was blocked for 1 h with TBST containing 5% powdered milk and was incubated with rabbit anti-CAR N-terminus primary antibody (abcam; Cat.-No. ab189216; 1:5000) in TBST (25 mM Tris-HCl, 500 mM NaCl, 0.5% Tween 20, pH 7.5) overnight at 4 °C and 4 h at room temperature. After washing, anti-rabbit HRP antibody (GE Healthcare, Chicago, IL, USA; Cat.-No. NA934; 1:5000) was added for 1 h at room temperature. After washing again, protein bands were visualised with ECL substrate (Biozym, Hessisch Oldendorf, Germany; Cat.-No. 541015). Membrane was stripped by two 10 min washing steps with stripping buffer (200 mM glycine, 3.5 mM SDS, 1% Tween 20 in H_2_O, pH 2.2), followed by two washes with PBS and two washes with TBST. Then, membrane was blocked again and reprobed with anti-His tag HRP antibody (abcam; Cat.-No. ab1187; 1:2000) in TBST for 1 h at room temperature.

### Sample preparation for MS

Protein bands were cut from the colloidal-blue-stained gel and destained by washing with 30% acetonitrile (ACN) in 100 µM ammonium bicarbonate buffer. Gel pieces were dried in a vacuum concentrator and digested with trypsin (Promega, Madison, WI, USA, Cat.-No. V5280) at a final concentration of 10 ng/µl in 3 mM Tris–HCl buffer, pH 8.8 overnight at room temperature and 200 rpm. Digestion was stopped by adding 1% trifluoroacetic acid (TFA). Gel pieces were incubated in LC–MS grade water with 0.1% TFA and 50% ACN for 45 min at room temperature and shaking at 400 rpm, and supernatant with extracted peptides was collected. This step was repeated once. Samples were dried in a vacuum concentrator and resuspended in 10 μl of LC–MS grade water with 0.1% TFA and 2.5% ACN. Particles were removed by high-speed centrifugation at 13,300× g and 4 °C for 5 min. nanoLC-MS/MS was performed with three independently prepared in-gel digestion samples.

### nanoLC-MS/MS and data analysis

nanoLC-MS/MS was performed as in [24] with some changes. Briefly, peptides were separated by an UltiMate 3000 RSLC Dionex system (Thermo Fisher Scientific, Dreieich, Germany). They were desalted on an Acclaim PepMap™ 100 C18 pre-column cartridge and separated on a 25-cm Acclaim™ PepMap™ 100 C18-LC-column (both Thermo Fisher Scientific). Effective gradient (15 or 35 min) was 4–30% or 4–35% solvent B (80% ACN, 1% TFA) with a flow rate of 300 nl/min. Online ESI-Orbitrap mass spectrometry measurements were carried out by a Q Exactive Plus instrument (Thermo Fisher Scientific) in data dependent top 10 acquisition mode. MS scan range was 350–2000 m/z with a resolution of 70,000 and the dynamic exclusion time of precursors for MS/MS was set to 5 or 15 s. Fragment ions were scanned with a resolution of 17,500 and fragmented with normalised collision energy of 28.

Peptide identification was performed with Proteome Discoverer 2.4 (Thermo Fisher Scientific). The amino acid sequence of human CAR’s extracellular domain (Uniprot P78310, amino acids 20-237 with C-terminal 6xHis-tag) as well as human neutrophil elastase (Uniprot P08246) were used as template for peptide spectrum matching. Furthermore, the randomly shuffled sequences (Sequence Manipulation Suite Version 2, Paul Stothard, 2004) were added as negative control for unspecific findings. Semi-tryptic digest was chosen with maximum two missed cleavage sites. Oxidation of methionine and N-terminal acetylation were set as variable modifications. For PNGaseF treated samples, deamidation of asparagine and glutamine was included as variable modification. False discovery rate (FDR) of 0.01 was selected and a minimum of two peptide spectrum matches (PSMs) was set as stringency filter. Peptide search was performed separately for deglycosylated and glycosylated samples, respectively, and results were merged in one consensus file.

### Treatment of cells with NE and determination of CAR surface levels by flow cytometry and immunofluorescence staining

Adherent CHO-CAR, HeLa, or SW13 cells were harvested using 500 µM EDTA in PBS and 2.5 × 10^5^ cells were resuspended in Ham’s F-12 medium containing 100 ng/µl NE. As control, NE reconstitution buffer was added instead of NE. Cells were incubated at 37 °C and 600 rpm for the times indicated in the text. Then, cells were pelleted and washed with PBS. Some samples were reduced by adding 50 mM DTT in PBS for 15 min at room temperature, followed by a washing step with PBS. Cells were either subjected to flow cytometry or to immunofluorescence microscopy.

For flow cytometry, cells were stained for CAR with E1-1 PE-labelled antibody (SantaCruz, Dallas, TX, USA; Cat.-No. sc-56892) diluted 1:66 in PBS for 1 h at room temperature. Cells were then washed and resuspended in PBS. Flow cytometry was performed with a Cytomics FC500 (Beckman Coulter, Brea, CA, USA) and CAR levels were determined as geometric mean of fluorescence intensity. To exclude bias, cell debris as well as cell doublets were excluded from the analysis by gating.

For immunofluorescence microscopy, cells were stained with rabbit anti-CAR antibody RmcB (Merck; Cat.-No. 05644) diluted 1:200 in Ham’s F12 medium for 1.5 h at 4 °C. After washing with PBS, cells were fixed with methanol for 10 min at − 20 °C and spotted on collagen-coated cover slides. Secondary goat anti-rabbit Cy3-conjugated antibody (JacksonImmunoResearch, West Grove, PA, USA; Cat.-No. 115165068) was diluted 1:1000 in 1% BSA in PBS and added to cells for 2 h at room temperature. Nuclei were stained with DAPI (CarlRoth, Karlsruhe, Germany; Cat.-No. 6335) at a 1:10^6^ dilution in 1% BSA in PBS for 5 min at room temperature. Samples were cover-slipped with Dako fluorescent mounting medium (Agilent, Santa Clara, CA, USA). With this method, only extracellular and not intracellular proteins are stained, which was confirmed using an antibody against intracellular vimentin (data not shown). Images were acquired with a TCS SP8 laser scanning confocal microscope (Leica, Wetzlar, Germany) using a 63 × oil immersion lens. Per image, 825 µm ﻿× 825 µm were scanned with automatic focussing on the DAPI channel. Fluorescence intensities were calculated with LasX software (Leica) as arithmetic means of determined grey-scale values.

### Statistics

Results are presented as mean ± SD. Statistical analyses were performed with GraphPad Prism software 8.3.0 (San Diego, CA, USA).

## Results

### Recombinant extracellular CAR domain is cleaved by MMP-3 and serine proteases

Recombinant human CAR extracellular domain (amino acids 20-237) with C-terminal 6xHis tag (rhECD) was digested by serine proteases and catalytic MMP domains overnight and cleavage products were visualised by Western blots using anti-CAR N-terminus antibody (abcam; Cat.-No. ab189216). As negative controls, rhECD was incubated with protease reconstitution buffer instead of the purified protease. General activity and specifity of proteases was confirmed with standard artificial substrates and inhibitors (data not shown).

**Fig. 1:**
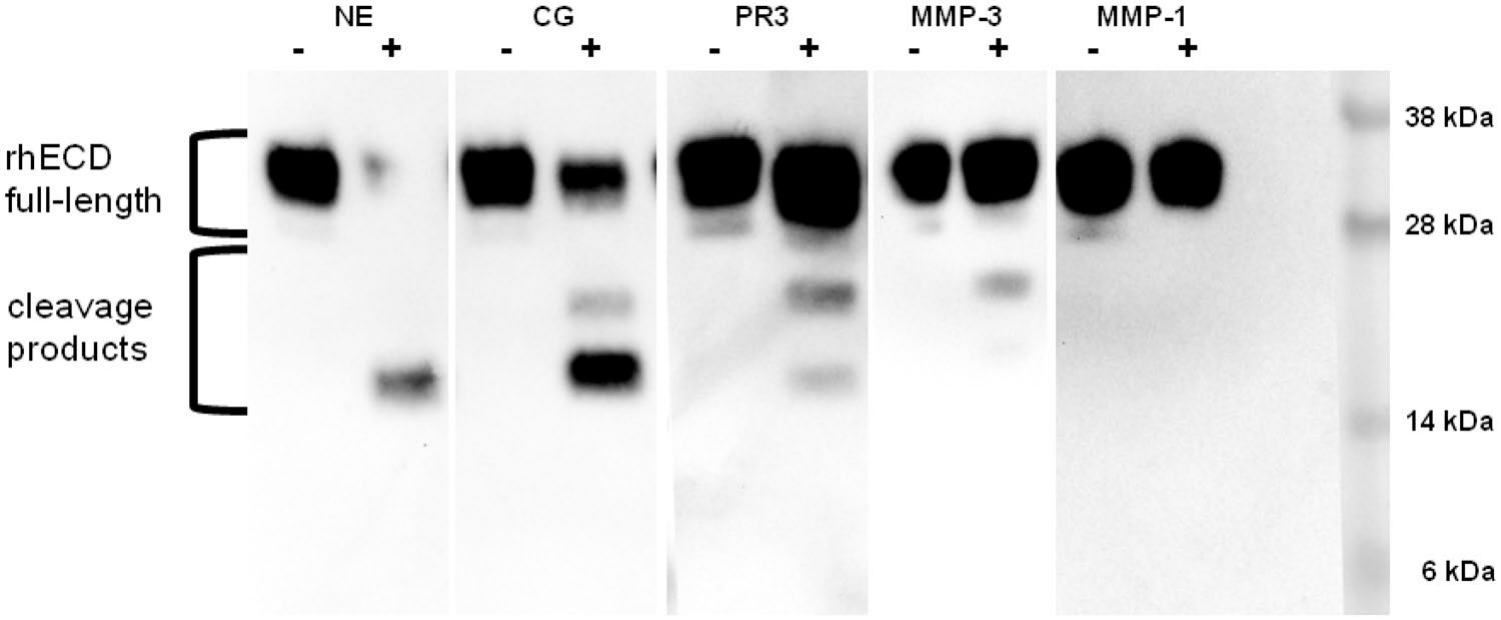
Human CAR recombinant extracellular domain (rhECD) is cleaved by serine proteases [neutrophil elastase (NE), cathepsin G (CG), proteinase 3 (PR3)] and matrix metalloproteinase 3 (MMP-3), but not MMP-1. rhECD was treated with proteases at a concentration of 100 ng/µl overnight at 37 °C and visualised with an anti-CAR N-terminus antibody (abcam; Cat.-No. ab189216) in Western blot. As negative control, protease reconstitution buffer was added instead of the protease. Distinct cleavage pattern with smaller products are visible

Undigested CAR rhECD runs at a height of about 30 kDa in PAGE (Fig. 1). An additional band of about 28 kDa probably represents a glycoform of rhECD as it disappears upon deglycosylation (data not shown). MMP-1 and other MMPs do not cleave rhECD (Fig. 1 and Online Resource 1). PR3, CG, NE, and MMP-3 digests result in smaller cleavage products of rhECD (Fig. 1).

**Fig. 2:**
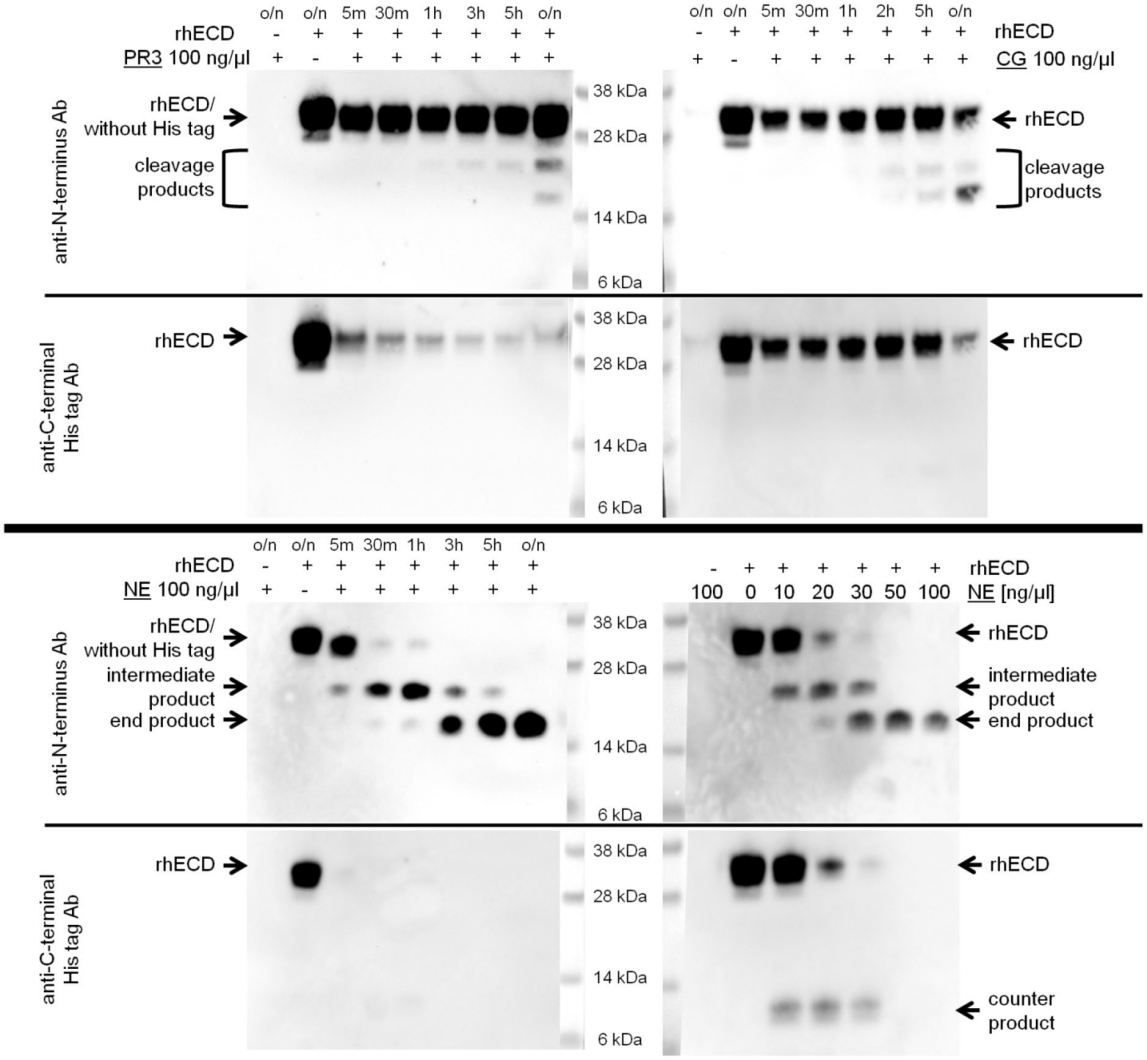
Recombinant human CAR extracellular domain (rhECD) is cleaved by serine proteases [proteinase 3 (PR3), cathepsin G (CG), neutrophil elastase (NE)] in a time- and dose-dependent manner. For time series (top panel for PR3 and CG and bottom panel left for NE), rhECD was treated with proteases (100 ng/µl) for different periods of time [5 min to overnight (o/n)] at 37 °C. As negative control, protease reconstitution buffer was added instead of the protease. For NE concentration series (bottom panel right), rhECD was treated with NE overnight at concentrations ranging from 0 to 100 ng/µl. Western blots were probed with anti-CAR N-terminus antibody (abcam; Cat.-No. ab189216), stripped, and reprobed with anti-C-terminal His tag antibody (abcam; Cat.-No. ab1187). PR3 and CG digests result in two cleavage products that include the N-terminal part of the protein and are not detected by the anti-C-terminal His tag antibody. However, reaction takes at least two hours and is not complete after overnight incubation. NE treatment results in an intermediate product (about 22 kDa) and an end product (about 17 kDa). Intermediate and end products are also observed, when NE is added in lower concentrations overnight. Those cleavage products comprise the N-terminal part of the protein and are not detected by the anti-C-terminal His tag antibody. A counter product (about 9 kDa) of the intermediate product is detected by anti-C-terminal His tag antibody at lower NE concentrations

Time and concentration series were performed with the serine proteases PR3, CG, and NE. Blots were probed with an anti-CAR N-terminus antibody raised against amino acids 20-50 (abcam; Cat.-No. ab189216), stripped, and reprobed with an antibody against the C-terminal His tag of rhECD (abcam; Cat.-No. ab1187). CAR rhECD is cleaved by those serine proteases in a time-dependent manner (Fig. 2). CG and PR3 digests of rhECD need long incubation times of hours, whereas NE digest is visible already within 5 min. All three serine proteases cleave CAR’s ECD at its membrane-proximal part as the cleavage products are recognised by anti-CAR N-terminus antibody, but not by anti-His tag antibody. PR3 and NE cleave next to the His tag already within 5 min resulting in a product that is recognised by anti-CAR antibody, but not anti-His tag antibody and runs at about the same height as full-length rhECD. The peptide with the His tag was not visible in the blot probed with the anti-His tag antibody. We suggest that it ran out of the gel due to its predictive size of about 1 kDa and the gel’s separation range of 160–3.5 kDa.

Since NE processes CAR rhECD much faster than CG and PR3, we considered it to be physiologically more relevant and focused on NE for subsequent investigations. For the same reason, MMP-3 cleavage was not further examined. NE cleavage pattern consists of two distinct products that we designated intermediate (about 22 kDa) and end product (about 17 kDa). Those products also occur when lower NE concentrations were used for overnight digest. At lower concentrations, NE does not cleave off the His tag. Therefore, the intermediate product has a C-terminal counter product (about 9 kDa) that is recognised by anti-His tag antibody (Fig. 2).

### NE cleaves recombinant extracellular CAR domain at its membrane-proximal Ig-domain D2

**Fig. 3:**
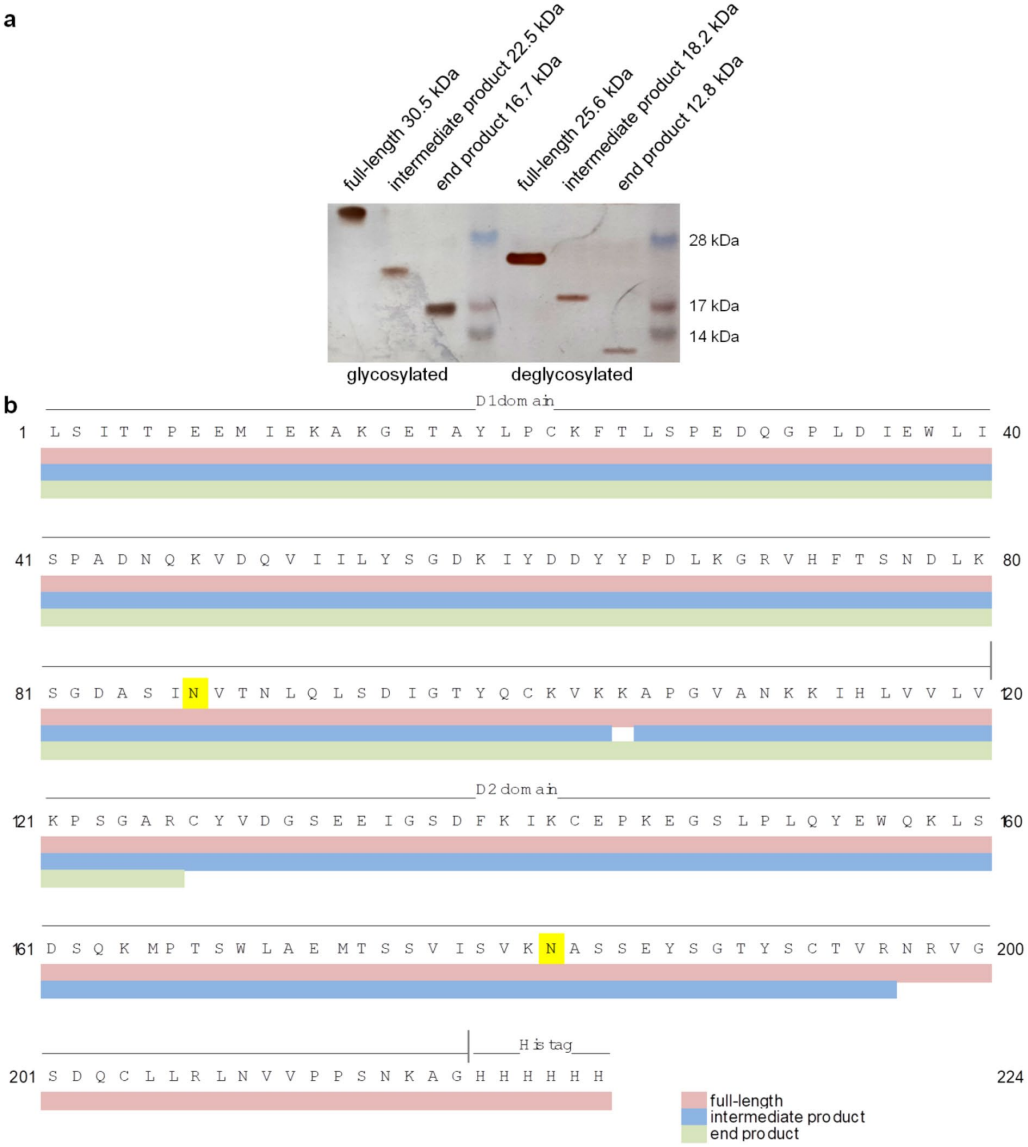
Neutrophil elastase (NE) cleavage sites in recombinant human CAR’s extracellular domain (rhECD) are located in the D2 domain. rhECD was treated with NE at a concentration of 100 ng/µl overnight at 37 °C, deglycosylated with PNGaseF, and visualised with PAGE. **a** Gel was silver-stained and molecular weights of the cleavage products were estimated from their running behaviour in the gel. Deglycosylation of digestion products allows size estimation without sugar moieties. **b** Protein bands were cut from a colloidal blue-stained gel, tryptically digested, and analysed with nanoLC-MS/MS. Data sets from glycosylated and deglycosylated samples were merged and identified peptides were mapped to the amino acid sequence of rhECD. Analysis of full-length rhECD (red) resulted in complete sequence coverage. The intermediate product (blue) contains the complete membrane-distal D1 domain and a large part of the membrane-proximal D2 domain. The end product (green) consists of D1 domain and a small part of D2 domain. N-glycosylation sites are marked in yellow. (Color figure online)

Full-length rhECD, intermediate and end product of NE digest were analysed in their glycosylated as well as deglycosylated forms by nanoLC-MS/MS (Fig. 3). Identified peptides are listed in Online Resource 2. Molecular mass of protein sequences covered by identified peptides were determined using ExPasy online tool and running behaviour of rhECD products in the gel was determined using LabImage. The silver-stained gel shows the optimal signal-to-noise ratio in order to visualise full-length rhECD, intermediate and end product as the most prominent bands (Fig. 3a).

Decrease in apparent molecular weight upon deglycosylation (Fig. 3a) corresponds with the observation that one glycosylation site in CAR results in an increase of apparent molecular weight in the gel of about 3 kDa [25, 26]. Loss of two sugar moieties is detected for full-length rhECD and the intermediate product, but not for the end product, which contains only one glycosylated asparagine in its sequence (compare location of asparagines in the amino acid sequence in Fig. 3b). Peptide identification with nanoLC-MS/MS (Fig. 3b) results in total coverage of rhECD (coloured in red, calculated mass is 24.8 kDa, runs at 25.6 kDa in the gel when deglycosylated). Deglycosylated intermediate product runs at 18.2 kDa in the gel and sequence coverage results in a product of 21.7 kDa (coloured in blue). For the end product, which runs at a height of 12.8 kDa in the gel when deglycosylated, sequence coverage yields 13.9 kDa (coloured in green). Therefore, both NE cleavage sites are located in CAR’s membrane-proximal immunoglobulin domain D2.

### NE treatment reduces CAR surface levels on mammalian cells

Proteolysis of CAR expressed on epithelial cells was investigated with CHO-K1 cells stably expressing human CAR (CHO-CAR; ovary) and human cell lines HeLa (cervix) and SW13 (adrenal gland) that express CAR endogenously. Cells were treated with NE or reconstitution buffer alone for different periods and CAR surface expression levels were monitored with either flow cytometry or immunofluorescence staining. Cells show different behaviour towards protease treatment. Whereas CHO-CAR and SW13 cells are still intact after 5 h NE treatment, HeLa cells show obvious cell damage within shorter incubation times (data not shown). Therefore, incubation times were adjusted accordingly.

**Fig. 4:**
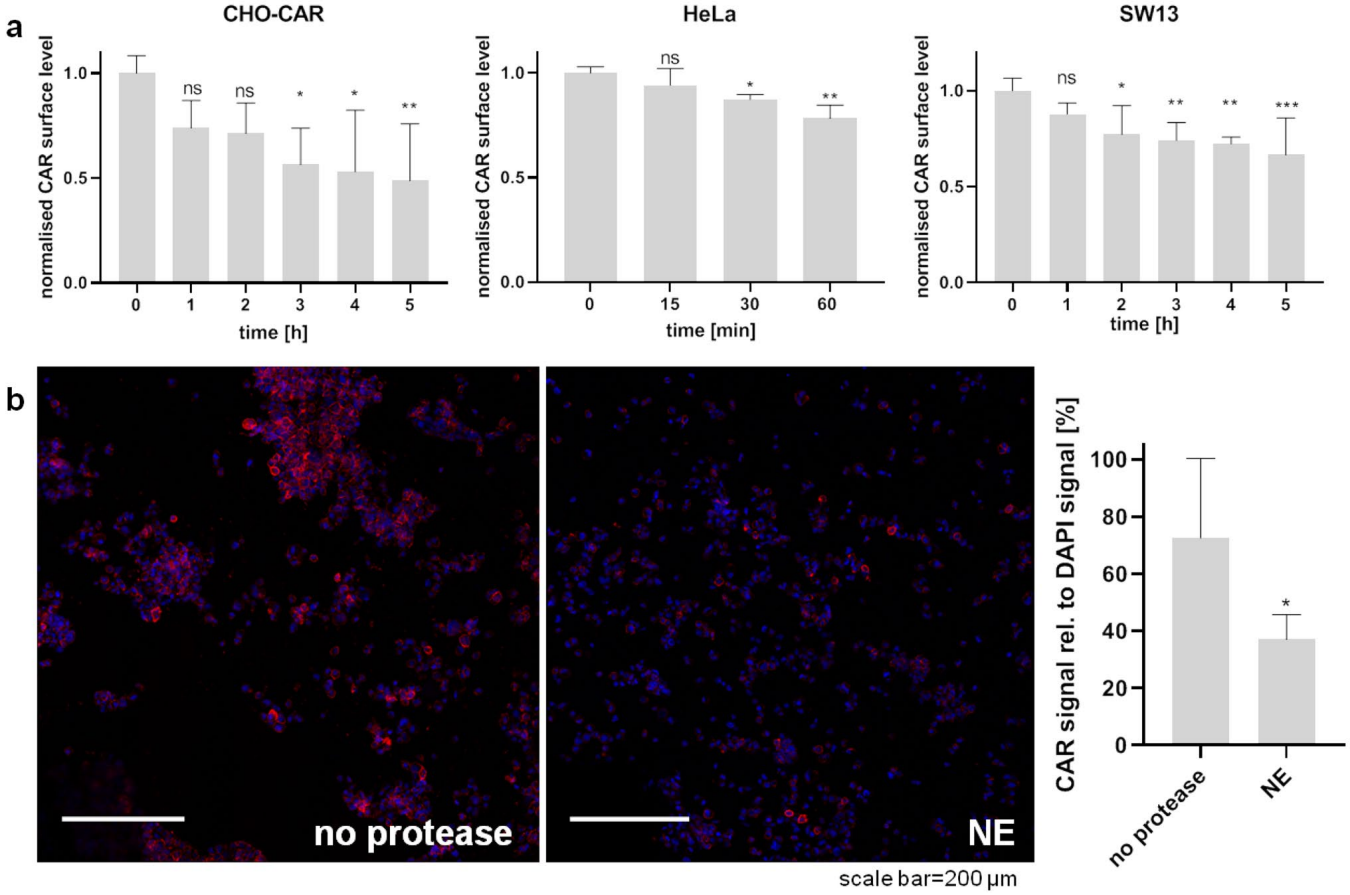
Neutrophil elastase (NE) treatment of epithelial cells results in decreased CAR surface levels. Cells were treated with NE (100 ng/µl) for different periods of time and CAR surface levels were visualised with antibodies against CAR N-terminus either by flow cytometry (SantaCruz; Cat.-No. sc-56892) (**a**) or immunofluorescence microscopy (Merck; Cat.-No. 05644) (**b**). **a** CAR surface levels of NE-treated cells were normalised to CAR surface levels of untreated cells and time point t = 0 was set as 1. CAR surface levels decrease significantly upon NE treatment in a time-dependent manner (ANOVA with multiple comparisons). Experiment was repeated three times (HeLa), four times (CHO-CAR), or five times (SW13). **b** CAR surface levels in CHO-CAR cells decrease significantly upon 3 h NE treatment as determined by fluorescence microscopy (paired *t* test). CAR signal (red) was set in relation to DAPI signal (blue). Two images were taken per treatment condition and experiment was repeated three times. Images are representative. (Color figure online)

CAR levels decrease in a time-dependent manner upon NE treatment as determined by flow cytometry (Fig. 4a). Reducing cell surface proteins with DTT after NE treatment does not alter the time course of CAR surface level decrease (Online Resource 3). This indicates that CAR fragments do not remain attached to the cell by disulfide bonds. A significant decrease of CAR surface protein level in CHO-CAR cells after 3 h NE treatment is also observed by immunofluorescence microscopy (Fig. 4b).

## Discussion

Here, we characterise human CAR as a potential target molecule for shedding mediated by NE. NE cleaves CAR’s ECD already within 5 min in vitro. For overnight digestion, a concentration of 10 ng/µl NE is sufficient for proteolysis. We suggest that these may be a physiologically relevant time span and concentration range, because the release of neutrophil granules results in high protease concentrations in micro-environments that are shielded from protease inhibitors (reviewed in [27]).

Our mass spectrometry analysis of CAR proteolysis by NE demonstrated cleavage within CAR’s membrane-proximal D2 domain. The exact cleavage sites of NE in human CAR were not determined, as identified peptides only cover the last trypsin cleavage site nearby. As NE prefers small, hydrophobic residues [28], potential cleavage sites would be V148 for the intermediate product and V218 for the end product (numeration according to full-length CAR sequence; in Fig. 3 those would be V129 and V199, respectively). Both cleavage products comprise complete D1 domain, which is expected to bind to Coxsackieviruses and adenoviruses [29, 30].

NE treatment decreases CAR surface levels of mammalian cells in a time-dependent manner as determined by flow cytometry and immunofluorescence staining. We cannot exclude the possibility that this is not due to shedding but a consequence of internalisation or degradation of CAR.

Shedding of human CAR by soluble NE may be a rapid mechanism to inhibit viral entry during ongoing virus infections. Membranous proteases ADAM10 and ADAM17 shed CAR’s ECD with subsequent decrease of adenoviral infection in the case of ADAM17 [21, 22]. A soluble protease like NE can be released at locally high concentrations from infiltrating neutrophils and would probably have more profound and faster effects on the myocardial tissue than membranous ADAM10 or ADAM17 that are transcriptionally regulated.

In previous works, other soluble proteases were investigated for their ability to process CAR or influence virus infections: HeLa cells treated with trypsin remained CVB-susceptible, probably because CAR fragments remained attached to the cells through disulfide bonds [31]. However, trypsin cleaved CAR’s ECD into fragments that could be visualised under reducing conditions [31] and high trypsin concentrations reduced CAR surface levels on Caco-2 cells [32]. In our study, we therefore did not harvest cells with trypsin and tested whether NE-digestion of cellular CAR was influenced by reducing conditions. DTT had no strong impact on CAR-level decrease (Online Resource 3), suggesting that NE cleavage products did not adhere to the cells via disulfide bonds.

Elastase, pancreatin, and chymotrypsin treatment of HeLa cells inhibited CVB attachment, which provided indirect evidence for a possible CAR-shedding [33, 34]. Recently, it was shown that bacterial cysteine proteases also process CAR in a highly specific manner [35], indicating that CAR shedding might be a process that is relevant for both host and pathogen.

In addition to myocarditis, Coxsackieviruses and adenoviruses can cause other inflammatory diseases like pancreatitis, pneumonia, gastrointestinal infections, and pericarditis [5, 6]. In all those diseases, CAR shedding may inhibit virus entry through three different mechanisms: (1) Reduced virus receptor levels correlate with cells’ susceptibility to virus infections [16, 17]; (2) CAR’s soluble extracellular domain may act as a virus trap as was shown for recombinant CAR proteins in in vivo models [18, 19, 36]; and (3) CAR’s soluble domain may block membranous unshed CAR for viruses by forming homodimers [26, 37, 38].

However, CAR shedding may not only have positive, but also detrimental effects for the host. CAR-JAML heterodimer interactions are important for neutrophil migration [39] and the presence of neutrophils increased adenovirus infections of polarized epithelial cells [40]. Therefore, CAR shedding by neutrophil proteases might be a regulatory mechanism of the immune reaction. Furthermore, cardiomyocyte-specific CAR knockdown in adult mice resulted in AV-block [41, 42] and low CAR levels were associated with arrhythmia vulnerability, cardiac conduction failure, and failed recovery from infarction in patients [43–46]. Besides, CAR may act as a pathfinder protein after cardiac injury as increased CAR levels were described in a rat infarction model [47] and in patients suffering from myocarditis and dilated cardiomyopathy [46, 48]. Therefore, CAR shedding might also decrease tissue regeneration ability or impair cardiac conduction.

Coxsackieviruses and adenoviruses use the transmembrane proteins decay accelerating factor (DAF) and integrins as co-receptors, respectively [49, 50]. Interestingly, recombinant human integrin ανβ5 is cleaved by NE already within five minutes, while cleavage of recombinant human DAF takes overnight incubation in vitro (data not shown). Adenovirus infections may therefore be influenced by shedding of CAR and integrins.

In summary, we showed that CAR might be a target for neutrophil elastase-mediated shedding with in vitro assays. We observed specific cleavage of CAR and proteolysis of the virus receptor on the cell surface. After confirmation of those findings in an animal model, CAR shedding and the release of a putative antiviral CAR fragment would define a novel mechanism of viral entry inhibition.

### Limitations

Our study investigates CAR shedding using in vitro experiments. However, CAR’s location at cell–cell contacts and the presence of protease inhibitors in vivo may hamper proteolysis by secreted neutrophil proteases. Future studies should address the actual physiologic conditions in which the host’s immune system and viruses interact directly with each other. Those may comprise experiments using other cell lines for example from lung or intestine, co-culture experiments with neutrophils and CAR-expressing cell lines, and virus infection experiments in cell culture and animal models.

## Supplementary Information

Below is the link to the electronic supplementary material.

Supplementary file1 (TIF 475 KB)—Matrix metalloproteinases (MMP) -2, -7, -8, -9, -10, -11, -12, and -13 catalytic domains do not digest recombinant human CAR extracellular domain (rhECD) after overnight incubation. rhECD was treated with MMPs overnight at a concentration of 100 ng/µl at 37 °C and visualised with an anti-CAR N-terminus antibody in Western blot (abcam; Cat.-No. ab189216). As negative control, protease reconstitution buffer was added instead of the protease

Supplementary file2 (XLSX 14 KB)-Peptides identified by nLC-MS/MS. rhECD was digested with NE (100 ng/µl) and some samples were deglycosylated by PNGase F. Full-length rhECD, intermediate and end products were cut from the gel and digested with trypsin. Peptides were analysed by nanoLC-MS/MS and data sets were merged for glycosylated and deglycosylated samples. For each peptide, its position in the protein sequence, its modifications, and the number of peptide spectrum matches (PSMs) are listed

Supplementary file3 (TIF 130 KB)—CAR surface levels of CHO-CAR and SW13 cells decrease by NE digest, when cells are kept under reducing conditions after NE treatment (ANOVA with multiple comparisons). CHO-CAR and SW13 cells were treated with NE (100 ng/µl) for different time points and DTT was added for 15 minutes after protease treatment to reduce proteins on the cells´ surface. Experiments were repeated three times

## Data Availability

The datasets generated during and/or analysed during the current study are available from the corresponding author on reasonable request.

## Abbreviations

CAR: Coxsackie- and adenovirus receptor
CG: Cathepsin G
MMP: Matrix metalloproteinase
NE: Neutrophil elastase
PR3: Proteinase 3
rhECD: Recombinant human CAR extracellular domain

## Materials availability

The datasets generated during and/or analysed during the current study are available from the corresponding author on reasonable request.
