# Supplementary figures and images for: Human Coxsackie- and adenovirus receptor is a putative target of neutrophil elastase-mediated shedding
Source: Mol Biol Rep. 2022 Feb 5;49(4):3213–23. doi: 10.1007/s11033-022-07153-2 (PMC8924087; doi:10.1007/s11033-022-07153-2)

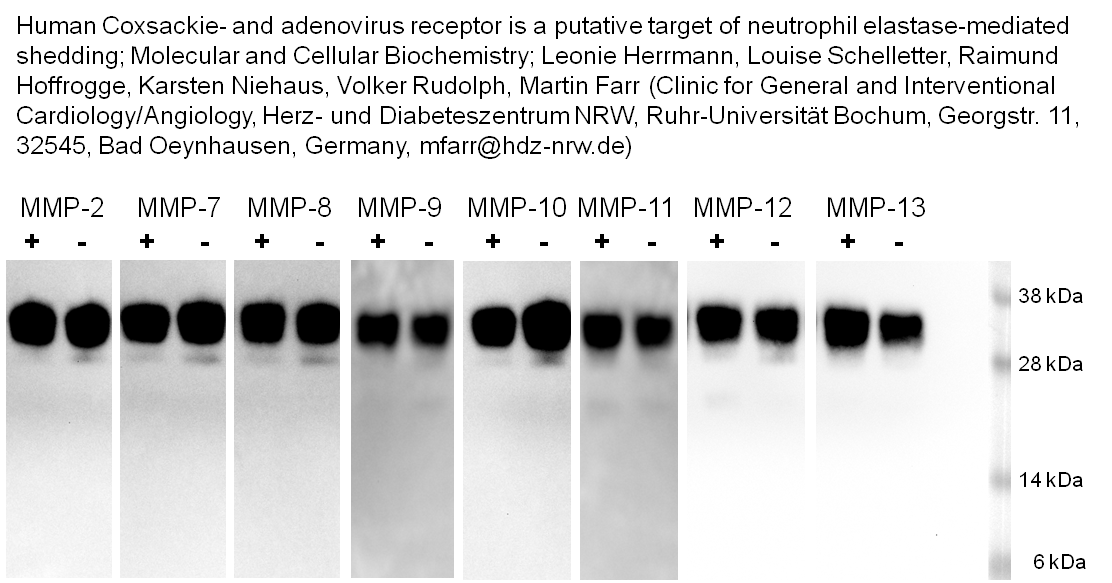

Supplement: Supplementary file 1 — Supplementary file1 (TIF 475 KB)—Matrix metalloproteinases (MMP) -2, -7, -8, -9, -10, -11, -12, and -13 catalytic domains do not digest recombinant human CAR extracellular domain (rhECD) after overnight incubation. rhECD was treated with MMPs overnight at a concentration of 100 ng/µl at 37 °C and visualised with an anti-CAR N-terminus antibody in Western blot (abcam; Cat.-No. ab189216). As negative control, protease reconstitution buffer was added instead of the protease [file 11033_2022_7153_MOESM1_ESM.tif]

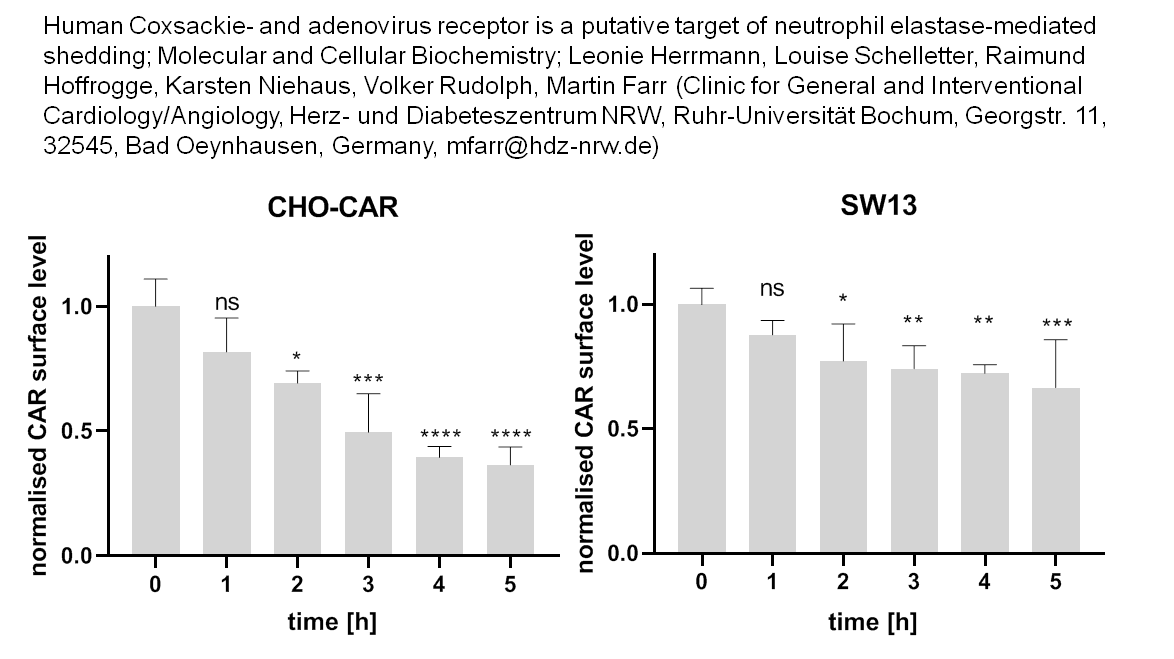

Supplement: Supplementary file 3 — Supplementary file3 (TIF 130 KB)—CAR surface levels of CHO-CAR and SW13 cells decrease by NE digest, when cells are kept under reducing conditions after NE treatment (ANOVA with multiple comparisons). CHO-CAR and SW13 cells were treated with NE (100 ng/µl) for different time points and DTT was added for 15 minutes after protease treatment to reduce proteins on the cells´ surface. Experiments were repeated three times [file 11033_2022_7153_MOESM3_ESM.tif]
